# The abundance of snail hosts mediates the effects of antagonist interactions between trematodes on the transmission of human schistosomes

**DOI:** 10.1186/s40249-024-01232-1

**Published:** 2024-09-10

**Authors:** Philippe Douchet, Bart Haegeman, Jean-François Allienne, Jérôme Boissier, Bruno Senghor, Olivier Rey

**Affiliations:** 1 IHPE Interactions Hotes-Pathogenes-Environnements, Centre National de La Recherche Scientifique, University of Montpellier, IFREMER, University of Perpignan Via Domitia, Montpellier, France; 2 Centre National de La Recherche Scientifique/Sorbonne Université, UMR7621, Laboratoire d’Océanographie Microbienne, Banyuls-Sur-Mer, France; 3 Institut de Recherche Pour Le Développement (IRD), Université Cheikh-Anta-Diop-IRD de Hann, 18524, BP 1386 Dakar, CP Senegal

**Keywords:** Biodiversity, Parasite, Hosts abundance, Trematodes, Schistosomiasis, Antagonistic interaction, Transmission

## Abstract

**Background:**

Combating infectious diseases and halting biodiversity loss are intertwined challenges crucial to ensure global health. Biodiversity can constrain the spread of vector-borne pathogens circulation, necessitating a deeper understanding of ecological mechanisms underlying this pattern. Our study evaluates the relative importance of biodiversity and the abundance of *Bulinus truncatus*, a major intermediate host for the trematode *Schistosoma haematobium* on the circulation of this human pathogen at aquatic transmission sites.

**Methods:**

We combined mathematical modelling and a molecular based empirical study to specifically assess the effect of co-infections between *S. haematobium* and other trematodes within their *B. truncatus* snail hosts; and *B. truncatus* abundance at transmission sites, on the production of *S. haematobium* infective cercariae stages released into the aquatic environment.

**Results:**

Our modelling approach shows that more competitive trematode species exploiting *B. truncatus* as an intermediate host at the transmission site level leads to higher co-infection rates within snail hosts, subsequently reducing the production of *S. haematobium* cercariae. Conversely, an increase in *B. truncatus* abundance results in lower co-infection rates, and a higher proportion of *S. haematobium* cercariae released into the environment. Our empirical data from the field support these findings, indicating a significant negative effect of local trematode species richness (*P*-value = 0.029; AIC = 14.9) and co-infection rates (*P*-value = 0.02, AIC = 17.4) on the dominance of S. *haematobium* based on our GLMM models, while *B. truncatus* abundance positively influences *S. haematobium* dominance (*P*-value = 0.047, AIC = 20.1).

**Conclusions:**

Our study highlights the importance of biodiversity in influencing the transmission of *S. haematobium* through the effect of antagonistic interactions between trematodes within bulinid snail hosts. This effect intensifies when *B. truncatus* populations are low, promoting co-infections within snails. In line with the One Health concept, our results suggest that maintaining high level of freshwater biodiversity to sustain global trematode diversity at transmission sites can help reducing the circulation of *Schistosoma* species locally.

**Graphical Abstract:**

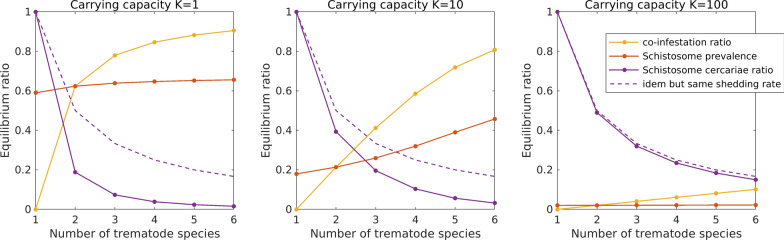

**Supplementary Information:**

The online version contains supplementary material available at 10.1186/s40249-024-01232-1.

## Background

One of the major consequences of global change is a drastic modification of the circulation of pathogens and an increase in disease emergences and outbreaks worldwide (e.g., dengue, chytridiomycosis, fasciolosis, schistosomiasis) [1–3]. The causes of these global-scale increases are multifactorial and the profound changes in biodiversity observed globally contribute significantly [4]. On one hand, while some populations/species are expanding, including humans and all domesticated species upon which human societies rely, an important amount of wildlife is in decline [5, 6]. On the other hand, it is generally assumed that parasite transmission increases with host density although generally not linearly [7]. As a result, the growth and densification of human and domestic animal populations contribute to the emergence and circulation of numerous pathogens sometimes causing severe health and economic issues which tend to extend at a planetary scale due to global international trades [8]. Moreover, an imbalance in the abundance of species assemblages in an existing community can favor certain competent host species to the detriment of many non-competent organisms, which, when present, mitigate the circulation of parasites through a dilution effect [9]. The generality and ecological importance of this dilution effect is, however, widely debated [10–12]. Additionally, host diversity in a community can support the co-existence of several circulating pathogen species, between which antagonistic (or beneficial) interactions within their hosts or vectors can significantly hamper (or facilitate) the circulation of least competitive pathogens [13].

Both facilitating and antagonistic interactions exist between trematodes. These parasitic worms display complex life cycles more generally including a vertebrate organism as definitive hosts and several successive invertebrates and/or vertebrate organisms are intermediate hosts including most of the time (aquatic) snails as first intermediate hosts. Such complex life cycles provide opportunities for interactions between trematode species and modulation of their transmission dynamics when coinfection occur especially in snail species that generally serve as amplifiers of trematodes in the environment. Some trematodes such as *Schistosoma* species are causative agents of severe forms of Human and livestock diseases. Interactions between *Schistosoma* species and other trematodes were historically documented with a focus on antagonistic interactions that could hamper their transmission as a mean of potential biological control strategies [14].

In fact, accruing evidence suggest that *Schistosoma* species that develop into sporocysts within their snail hosts are considered as moderate to bad competitors compared to other trematode species [13]. Moreover, other trematode species that develop into rediae within snail hosts can predate the co-occuring *Schistosoma* sporocysts under development while consuming snail tissues [15]. At natural transmission sites of *Schistosoma* species, the importance of such antagonist interactions on the circulation of *Schistosoma* species is generally underappreciated. Importantly however the snail intermediate hosts used by some *Schistosoma* species (e.g. *Biomphalaria* spp. and *Bulinus* spp.) generally host numerous trematode species hence providing opportunities for many intra-host interactions that involve *Schistosoma* species. For example, up to 29 trematode species have been identified in natural populations of *Biomphalaria pfeifferi* and *Bi. sudanica* established over a constrained geographical scale, these two snail species being important hosts for *Schistosoma mansoni* in eastern Africa [13]. Moreover, and as previously mentioned, *Schistosoma* species harbor low competitiveness compared to many other species of trematodes [13]. In line with these two characteristics, recent modelling approaches predict over 50% decrease in the transmission of *S. mansoni* in the presence of some highly competitive trematodes coinfecting *Bi. pfeifferi* – such as *Calicophoron sukari*, a widely distributed parasite across livestock [13].

As a simple baseline, the prevalence of coinfection in a host population harbouring two parasites equals the product of the prevalence of single infection of each parasite in the population [15]. However, coinfection rates observed *in natura* are generally lower than this theoretical expectation and several hypotheses might explain such departure. Indeed, interspecific competition leading to the rapid exclusion of one interacting species, a high cost of co-infection on hosts fitness, the recruitment heterogeneity either due to random or due to parasite attractance for hosts through chemotaxis, and immune priming, are mechanisms by which co-infection or their detection can be limited *in natura* [15, 16]. Intriguingly, the effect of host abundance on co-infection rates is generally neglected since this theoretical expectation relies on the relative prevalence of co-infection, which corresponds to the proportion of hosts that are infected (or co-infected) among all hosts present in the environment. However, according to the generally acknowledged density-dependent nature of pathogen transmission [17], it is expected that host density also influences the co-infection rates, the occurrence of antagonistic interactions and ultimately the transmission of less competitive parasites. If so, this could constitute an additional indirect mechanism by which host density is positively correlated with parasite transmission by reducing co-infections and a release from antagonistic interactions.

Using the *S. haematobium* – *Bulinus truncatus* system as a model, the objective of this study is to assess the relative importance of the density of *B. truncatus* at transmission sites and the diversity of trematodes that use *B. truncatus* on the risk of transmission of *S. haematobium* (Schistosomatidae). *S. haematobium* is a trematode species causing the urogenital form of bilharziasis in humans mainly in the tropical regions in Africa [18]. It displays a complex two-host lifecycle that involves humans as vertebrate hosts, in which the adults develop and reproduce sexually resulting in the production of thousands of eggs that are released in the environment through the urinary system of the host; and *B. truncatus* (Bulinidae), a freshwater snail host in which the parasite reproduce asexually resulting in the production of cercariae that are released in the aquatic environment and actively seek and penetrate the skin of a new vertebrate definitive host.

We hypothesise that for a given abundance of *B. truncatus*, an increase in the specific richness of trematodes using *B. truncatus* as a host at the community scale would lead to an increase in co-infection rates that involves *S. haematobium*. Given that *Schistosoma* species (including *S. haematobium*) tend to be moderate competitors [13], any increase in coinfection rates may reduce *S. haematobium* development and hence the overall risk of transmission if less cercariae are released. Moreover, an increase in the abundance of *B. truncatus* would lead to a reduction of the co-infection rates and the associated antagonistic interactions thus ultimately enhancing the risks of schistosomiasis transmission to Humans.

To test these hypotheses, we first built a simple mathematical model to describe (i) how the number of competitive trematodes using *B. truncatus* influence the amount of *S. haematobium* cercariae released in aquatic systems, and (ii) how snail density (here carrying capacity) influences co-infections rates and the associated antagonistic effects on the release of *S. haematobium* cercariae at the transmission site level. We confronted this mathematical model to an empirical field work study conducted in Northern Senegal at nine previously identified urogenital schistosomiasis transmission sites. We combined traditional malacological and parasitological approaches and next-generation molecular tools to empirically study the effect of the trematodofauna using *B. truncatus* as intermediate host and the local abundance of *B. truncatus* on the transmission of *S. haematobium*.

## Material and methods

### Mathematical model

To specifically assess the effect of snail host abundance and the effect of possible trematode-trematode antagonistic interactions on the production and emission of cercariae of *Schistosoma* species we built a mathematical model. We constructed a dynamical model of an aquatic ecosystem in which a snail population is infected by n trematode species, one of them being the *Schistosoma* species. Trematodes enter the system as miracidia, which can infect susceptible snails. Infected snails shed cercariae, which are taken up by mammal hosts. This leads to a model with four types of dynamical variables: the density of miracidia for the different trematode species, the density of susceptible snails, the density of snails infected by one or more (up to a maximum of n) trematode species, and the density of cercariae for the different trematode species. The general model becomes rapidly overwhelming with increasing trematode diversity. In the Additional file 1 we show that the number of variables increases exponentially with the number of trematode species n. To keep the model manageable, we applied the following assumptions:

- 1. We required that infected snails have a smaller growth rate and a larger death rate than non-infected snails. For simplicity, we implemented an extreme version of this requirement, and assumed that the growth rate of infected snails and the death rate of non-infected snails are both zero.
- 2. We assumed that snails infected by multiple trematode species only shed cercariae of a single trematode species. We also required that the *Schistosoma* species is less competitive, and translated this difference in competitivity as a lower shedding rate of *Schistosoma* cercariae. For simplicity we implemented an extreme version of this requirement, and assumed that snails that are co-infected by the *Schistosoma* species and one or more other trematode species never shed cercariae of the *Schistosoma* species.
- 3. We assumed that all trematode species, including the *Schistosoma* species, are equivalent, in the sense that they have the same trait values apart from the shedding rate (see previous assumption). Specifically, the input rate of miracidia is the same for all trematode species, as are the miracidia loss rate, the death rate of infected snails, the cercariae loss rate and the uptake rate of cercariae by mammal hosts.

In the Additional file 1 we express these assumptions in terms of the model parameters, and use them to derive a drastic simplification of the general model. Specifically, we show that the model equilibrium can be computed from a system with a number of variables that increases only linearly in the number of trematode species n.

We studied the equilibrium properties as a function of two model parameters: the number of trematode species and the carrying capacity of the snail population. When varying the number of trematode species, we assumed that the miracidia input rate per trematode species is independent of diversity, so that the total input rate (i.e., summed over all trematode species) increases proportionally with diversity. In particular, the input rate of *Schistosoma* miracidia is unaffected by the presence of other trematode species. We also assumed that the total shedding rate (i.e., the shedding rate per infected snail and summed over all shedded trematode species) is independent of the number of species infecting the snail. This implies that the shedding rate per snail infected by a particular trematode species decreases with trematode diversity, as some of these snails are co-infected with other trematode species. This holds especially for snails infected by *Schistosoma*, as co-infection by another trematode species causes the *Schistosoma* shedding rate to drop to zero (see third assumption).

We reported the simulation outcome using three variables: the co-infestation rate, defined as the density of infected snails by two or more trematode species divided by the density of infected snails by one or more trematode species; the *Schistosoma* prevalence, defined as the density of snails infected by the *Schistosoma* species divided by the total density of snails (infected or not); and the *Schistosoma* cercariae ratio, defined as the density of *Schistosoma* cercariae divided by the total density of cercariae.

In the Additional file 1 we provide an in-depth description of the model equations and our simulations. We also provide the Matlab R2024a code (MathWorks, inc., Natick, USA) for all reported model simulations (Additional file 2 & 3).

### Empirical field work

#### Study sites description and ethical consideration

**Fig. 1:**
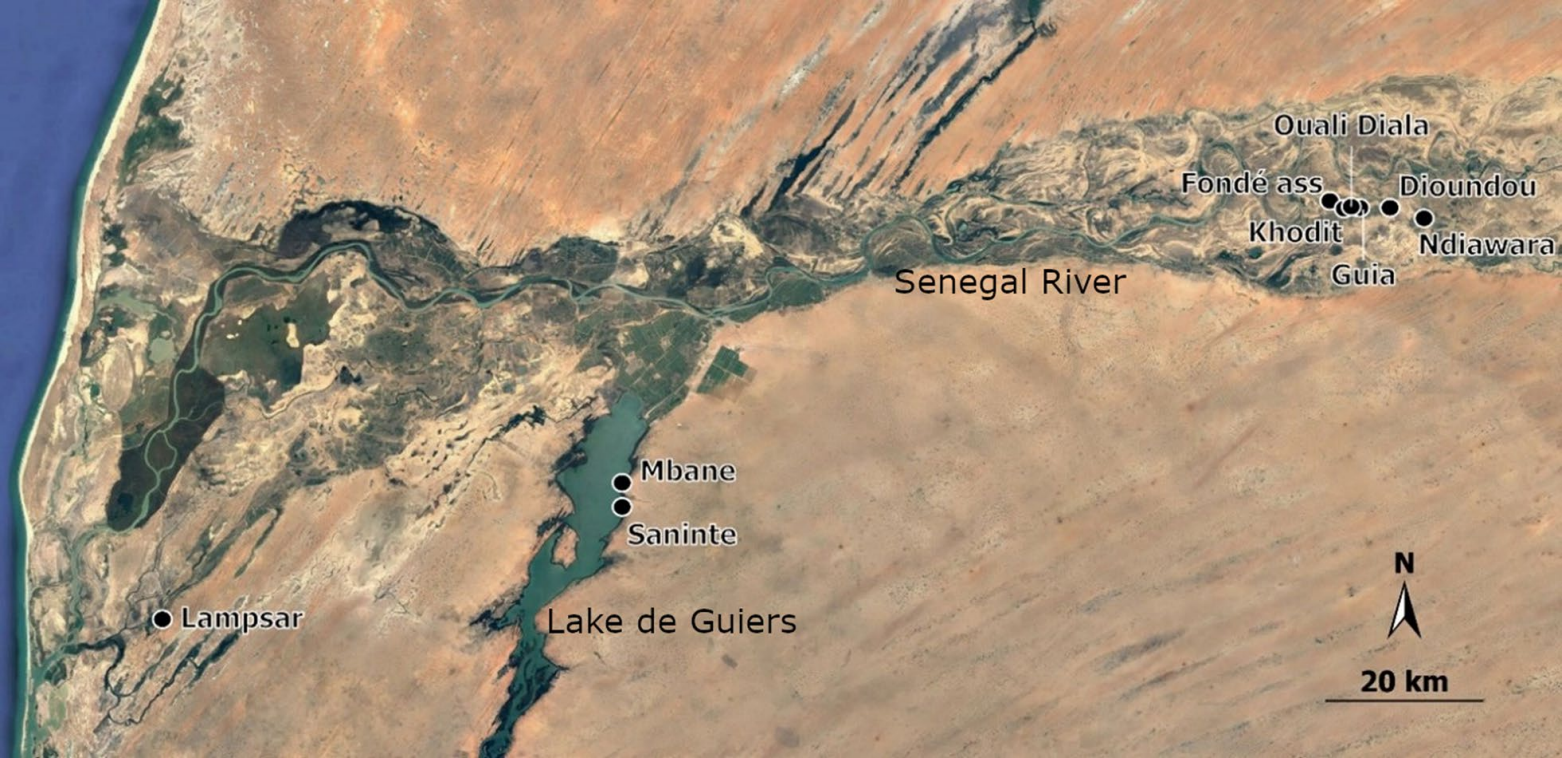
Satellite map of the studied area in Northern Senegal. This map indicates the location of the nine targeted sites (black dots) and the name of the nearby nine villages associated to these *Schistosoma haematobium* transmission sites

An empirical fieldwork study was carried out in the region of the Senegal river basin during the dry season in February 2022 (Fig. 1). We targeted nine aquatic sites previously identified as potential *S. haematobium* transmission sites that differ from an ecological point of view (i.e. river, irrigation canal and lake) and hence possibly harbouring different trematode communities. Indeed, each of these transmission sites is close to a village where the prevalence and intensity of urogenital schistosomiasis among children was previously reported [19].

The transmission sites close to the villages of Ndiawara (16°35′04″N / 14°50′58″W), Ouali Diala (16°35′56″N / 14°56′10″W), Dioundou (16°35′50″N / 14°53′22″W), Fonde Ass (16°36′20″N / 14°57′38″W) and Khodit (16°35′45″N / 14°56′42″W) are located in the middle valley along a tributary of the Senegal River (“le Doue” river). The transmission site close to the village of Guia (16°35′51″N / 14°55′31″W) is located along an irrigation canal that drains water from the river “le Doue”. The transmission sites nearby the villages of Mbane (16°16′15"N / 15°48′7"W) and Saneinte (16°14′32"N / 15°48′6"W) are located along the east shore of the lake de Guiers. The transmission site close to the village of Lampsar (16°6′34"N / 16°20′58"W) is in an inlet in the lower valley of the Senegal river delta (Fig. 1).

#### eDNA, snails and trematodes field sampling

To assess the abundance of *B. truncatus* and to identify all trematode species present at each of the nine studied sites as free-living stages, aquatic environmental DNA samples (eDNA) were collected using water filter capsules according to Douchet et al. (2022) [20]. Filtrations were carried out from the entire water column along the banks until the filter’s membrane clogged, and the volume filtered at each site was recorded. At each site but Khodit, 1.5 L of commercial spring water was filtered following the same protocol as a technical field negative control. Once the water filtrations completed, capsules were drained, filled with 50 ml of Longmire buffer solution to preserve eDNAs [21], vigorously shaken, and stored at room temperature and at dark until subsequent DNA extraction.

Once the eDNA sampling achieved, all freshwater snails found at each sampling site were systematically collected manually or by scooping the grass on the water bench using a colander for about 30 min to one hour. Snails were morphologically identified using a taxonomic key [22] and individualized on well plates filled with dechlorinated water and left to emit trematodes for two hours in the afternoon under natural sunlight. Cercariae from each emitting snail were collected and transferred onto FTA cards and stored at room temperature until DNA extraction and molecular identification. Coupled with eDNA samples, this approach allows a better characterisation of the trematode community at each transmission site and a quantification of the prevalence of each emitted trematode species among the locally established snail populations including *B. truncatus*. All snails morphologically identified as *B. truncatus* (either emitting or non-emitting) were preserved individually in alcohol until DNA extraction. Subsequent molecular analyses aimed at validating the species attribution of each *B. truncatus* individual, check for the presence and identification of potentially developing trematodes within each snail, refine measures of prevalence of each trematode species, and identify possible coinfections.

### Experimental infection

Twenty-six non-emitting snails (although possibly naturally infected by trematodes) sampled at sites Guia, Fonde Ass, Khodit and Saneinte were kept alive and individually exposed to three *S. haematobium* miracidia for two hours in 24 well plates filled with dechlorinated water. The miracidia used for this experimental mollusc exposition were from a homogenised pool of *S. haematobium* eggs of eight children from the Lampsar village provided by the Sen_Hybrid_Invasion project (see acknowledgment section). The 104 exposed snails (i.e., 26 × 4) were maintained at 24 °C in dechlorinated water under natural light and fed ad libitum for a period of one month. During this period, survival was checked daily. At the end of this period, the infection status of each *B. truncatus* was monitored by emission of cercariae once a week until the snails died. To this end, *B. truncatus* were individualized on clean well plates filled with dechlorinated water and stimulated to emit trematodes for 4 h in the afternoon under artificial light. Cercariae released from emitting snails were collected and stored at −20 °C until DNA extraction for subsequent molecular identification. All along the experiment (i.e., 4 months), each emitting snail was kept individualised to identify possible trematode species emission shifts through time. At the end of the experiment, all *B. truncatus* individuals (either emitting or non-emitting) were preserved in alcohol individually until DNA extraction.

### Molecular approaches

#### DNA extractions from samples

To extract eDNAs from water filtrates, the Longmire solution contained in each capsule was poured into three 50 ml tubes as technical replicates. For the field negative controls (i.e., Spring water filtrates), each capsule content was recovered in one 50 ml tube only. All tubes were centrifugated at 16,000 × *g* for 20 min and the supernatant was removed. We then collected 0.25 g to 0.5 g of sediment from the pellet from each tube or 500 µl of Longmire remaining at the base of the tube when not enough material was observed. For negative controls, 500 µl of Longmire were systematically retained when discarding the supernatant. This pre-extraction step led to the processing of 35 samples (i.e., eight negative controls and three extraction replicates for each of the nine environmental samples). Total environmental genomic DNA was extracted from each triplicate and negative controls using the Qiagen’s Dneasy PowerSoil Pro Kit following supplier recommendation performing the physical lysis with a MagNA Lyser at a speed of 7000 × *g* for 30 s.

DNAs from all cercariae obtained from the field survey, from the exposure experiment and from all *B. truncatus* snails were extracted using the Qiagen DNeasy Blood & Tissue kit (Qiagen, Hilden, Germany). Regarding cercariae preserved onto FTA cards, we manually isolated 0.5 cm in diameter pieces containing the biological material using a sterile punch and placed them in 1.5 ml tubes. Four samples consisting in 0.5 cm in diameter pieces from cards on which ultrapure water had been deposited were also isolated as DNA extraction negative controls. For cercariae preserved in 1.5 ml tubes stored at −20 °C, we thawed the tubes and centrifuged them at 20,000 × *g* for 10 min before removing the supernatant. To obtain extraction and PCR negative controls, we also centrifuged two tubes that contained ultrapure water following the same protocol. For *B. truncatus*, we first rinsed the snails with tap water and ground them entirely and individually with a sterile pestle in 1.5 ml tubes. To obtain extraction and PCR negative controls, we also processed seven assuredly non-infected *B. truncatus* from our laboratory collections in the same way. Moreover, three extraction/PCR positive controls were prepared from three additional non-infected laboratory *B. truncatus* individuals, to which one *S. haematobium* cercariae from our laboratory collections was artificially added before DNA extraction processing. These pre-extraction steps led to the processing of 328 samples and 16 controls (i.e., 29 FTA card pieces and four associated negative controls, 28 cercaria preserved in alcohol and two associated negative controls and 281 *B. truncatus* including 173 *B. truncatus* from the field and 98 from the experimental infection and three and seven associated positive and negative controls). From these samples, we then followed the Tissue protocol as recommended by the supplier applying a two-hour lysis for cercariae and an overnight lysis for snails.

#### Taxonomical assignment of trematode cercariae by barcoding

To taxonomically assign each cercariae emitted from snail hosts to a trematode species, DNAs extracted from cercariae of each emitting snail were SANGER sequenced at the *28S D2* rDNA gene domain (*28S*) and at the *16S* rDNA gene (*16S*) [20]. PCRs were run using the obtained 57 DNAs extracted from cercariae and the six negative controls on both markers. PCRs were performed using the GoTaq^®^ G2 Hot Start Polymerase kit of Promega (Promega, France). Each PCR reaction contained Colorless Buffer at 1 × , MgCl_2_ at 1.5 mmol/L, dNTPs at 0.2 mmol/L, primers at 0.4 µmol/L, 1.25 units of GoTaq G2 Hot Start, 2 µl of DNA sample, and ultrapure water for a total PCR reaction volume of 35 µl. For the *28S* marker, the PCR program was used as follows: An initial denaturation step at 94 °C for 3 min followed by 40 cycles with a denaturation step at 95 °C for 30 s, a hybridization step at 56 °C for 30 s and an elongation step at 72 °C for 30 s. We finally performed a final elongation step at 72 °C for 5 min. For the *16S* marker, the same PCR program was used, except that 35 cycles were performed, the hybridization temperature was 54 °C and the elongation time was 15 s. Ten microliters of the resulting PCR products were migrated on a 2% agarose gel for 20 min at 135 V and revealed using a Vilber Infinity 1000 imaging system (Vilber, France). Each individual PCR product from the 57 cercariae DNA extracts that displayed an expected theoretical size was then sequenced in both the forward and reverse directions on an ABI 3730xl sequencer (Thermo Fisher scientific, USA) at the GenoScreen platform (GenoScreen, Lille, France). The sequences generated were aligned, trimmed, and taxonomically assigned with a MEGABLAST analysis [23] for the *28S* and the *16S* markers. For the taxonomical assignment of each cercariae, the common taxonomic rank to all hits above 96% of identity over 97% of coverage were kept.

### Molecular taxonomic validation of *B. truncatus* and infection diagnostic

The 271 *Bulinus* spp. sampled in the field from which total DNA was extracted were diagnosed to verify that they belonged to the species *B. truncatus* using a molecular diagnosis by loop-mediated isothermal amplification (LAMP) based on the internal transcribed spacer (ITS2) *B. truncatus* species-specific [24]. DNAs extracted from *Bulinus* spp. were first diluted at 1/100e and a negative control consisting in a 1/100e diluted DNA extraction from *Bulinus globosus* was used. Each LAMP reaction contained an Isothermal Amplification Buffer II reaction buffer [20 mmol/L Tris-HCl, 10 mmol/L (NH4)_2_SO_4_, 150 mmol/L KCl, 2 mmol/L MgSO_4_, 0.1% Tween 20, pH 8.8 at 25 °C (New England Biolabs, UK)] at 1 X, additional MgSO_4_ at 3 mmol/L, dNTP at 1.0 mmol/L, internal primers FIP and BIP at 1.2 µmol/L, external primers F3 and B3 at 0.2 μmol/L, LOOP primers LB and LF at 0.4 µmol/L, 1 U of Bst 2.0 WarmStart DNA polymerase (New England Biolabs, UK), 1 µl of DNA sample, and ultrapure water for a total reaction volume of 10 µl as described in Blin et al., 2023 [24]. Reaction was performed in a thermocycler at 63 °C for 45 min followed by an enzyme inactivation phase at 80 °C for 5 min. Result visualizations were done using a final point visual detection of fluorescence after adding 1 μl of 1:50 diluted 10,000 × *g* concentration of SYBR Green (Invitrogen, USA) (green: positive = *B. truncatus* species; orange: negative = other than *Bulinus* species).

To detect trematodes within the tissues of each *B. truncatus*, we used the 16S rDNA gene metabarcode initially developed to characterize trematode communities from eDNA [20] as a molecular diagnostic tool. PCRs were performed using the GoTaq^®^ G2 Hot Start Polymerase kit of Promega (Promega, France) following the same PCR condition as described previously, except that reactions were performed in a final volume of 10 µl and using 2 µl of DNA extracted from *Bulinus* spp. diluted 1:100. Amplification success was assessed visually by migrating the ten microliters of the resulting PCR products on a 2% agarose gel for 20′ at 135 V and revealed using a Vilber Infinity 1000 imaging system (Vilber, France).

### Trematodes-specific MiSeq sequencing on the infected *B. truncatus* individuals and on the eDNA to characterize trematode communities

A total of 137 positive *16S* metabarcoding NGS libraries were prepared following the Illumina two-step PCR protocol, using the Trem_16S_F1 and the Trem_16S_R2 primers set up with Illumina adapters (Illumina, USA) using 2 µl of eDNA or DNA extracted from *B. truncatus* diluted at 1:100e as previously described [20]. Libraries were sent and paired end sequenced (2 × 250 bp) on an Illumina MiSeq™ at the BioEnvironnement platform (University of Perpignan Via Domitia, France). Three samples were eliminated from the analyses of the MiSeq sequencing because they did not meet the defined threshold of 25,000 sequences required to normalise samples by rarefaction (i.e., one non emitting *B. truncatus* from the site Ouali Diala, and two PCR duplicates from water eDNA).

### Abundance of *B. truncatus* determination by ddPCR on eDNA

Abundance of *B. truncatus* at each study site was assessed by digital droplet PCR (ddPCR) from eDNA sampled by water filtration according to [25]. We first pooled the eDNA extraction triplicates from each of the nine sampling sites. As negative control, we also pooled DNAs extracts from one individual of each mollusc species sampled during the field work in addition to DNAs extracts from three *B. truncatus* sister species (i.e., *B. globosus*, *B. senegalensis* and *B. umbilicatus*) from our laboratory collection. As positive control, we used a DNA extract at 0.01 ng/µl from one *B. truncatus* from our laboratory collection. We ran ddPCRs using the TaqMan technology on a QX200 AutoDG Droplet Digital System (BioRad, USA) at the Bio-environnement platform (Perpignan, France). We used the Btco2F (5′-ATTTTGACTTTTACCACCAT-3′) and Btco2R (5′-GATATCCCAGCTAAATGAAG-3′) primers combined with the FAM-labelled probe Btco2P (5′-TCGAAGGAGGGGTTGGAACAGG-FAM-3′). ddPCR reactions were performed using the BioRad ddPCR Supermix for Probes (No dUTP) (BioRad, USA). Each ddPCR reaction contained the BioRad MIX at 1 X, primers at 0.5 µmol/L, probes at 0.25 µmol/L, 7 µl of eDNA template or 2 µl of control DNAs templates, and ultrapure water for a total ddPCR reaction volume of 20 µl. After the droplets generation, the ddPCR program was used as follows: An enzyme activation step at 95 °C for 10 min followed by 40 cycles with a denaturation step at 94 °C for 30 s and an annealing/extension step at 60 °C for 60 s, with a ramp setting to 2 °C/s. We finally performed an enzyme deactivation step at 98 °C for 10′. The resulting fluorescence signal were analysed using QuantaSoft software V1.7 (BioRad, USA). A signal was considered positive (i.e., with the presence of *B. truncatus* DNA) if at least one positive droplet was detected and if the positive droplet displayed the same order of fluorescence magnitude as the positive droplet obtained from a ddPCR positive control.

### Data analysis

#### Characterisation of trematode communities present in the water and exploiting *B. truncatus* populations

The resulting amplicon sequence datasets from the MiSeq sequencing was processed using the Find Rapidly OTUs with Galaxy Solution (FROGS) [26] according to reference [20]. Briefly, the produced datasets were pre-processed by filtering out the sequences to keep amplicon sizes from 150 to 400 nucleotides. The remaining sequences were next clustered into operational taxonomic units (OTUs) using the swarm algorithm and using denoising and an aggregation distance of three [27]. The resulting dataset was filtered out for chimeras using VSEARCH [28]. Singletons and underrepresented clusters (i.e., clusters whose number of sequences were < 0.1% of the total number of sequences) were removed. Lastly, we conservatively considered that a given OTU was present in a library if its number of sequences was > 0.1% of the total number of sequences in this library.

Each OTU was next assigned to a taxonomic level (either a species or a genus) using a two-step BLAST affiliation process. The first BLAST analysis was computed using the standalone blastn program (NCBI, USA) contained in the *BLAST* + package and a custom trematode sequence database containing 174 sequences (82 sequences from the NCBI database and 92 sequences from a custom internal trematode sequence database including the sequences obtained from the amplicons generated by the SANGER sequencing on cercariae). The second BLAST analysis was performed using the online MEGABLAST tool and based on the non-redundant database without restricting parameters to achieve affiliation of OTUs that could not be assigned in the first BLAST analysis. OTUs were assigned to a species if the sequences presented a minimal blast coverage of 97% and a pairwise identity above 99% with the affiliated sequence. OTUs were assigned to a genus if the sequences presented a minimal blast coverage of 97% and a pairwise identity above 96% with the affiliated sequence. OTUs that could not be assigned to a species or genus were assigned to a higher taxonomic rank using a clustering method based on the pairwise genetic distances between the OTUs and the same set of 174 sequences as above. We then aligned these sequences with T-Coffee on EMBL-EBI [29] and built a neighbor joining phenetic tree based on the percentage of nucleotide differences from the obtained alignment using Jalview version 2.11.1.4 [30] for visualization.

Subsequent analyses were then performed on R version 4.3.1 (Lucent Technologies, Jasmine Mountain, USA). The significance levels of statistical tests were set at 0.05. Each library was normalized by rarefaction at 25,000 reads using the package *Vegan* version 2.6–4. The trematode composition, species number and relative abundance in each *B. truncatus* individual or water sample was next assessed using the package *phyloseq* [31]. We assessed the co-infection rates among infected *B. truncatus* and the number of trematode species involved in each co-infection. We also tested whether parasite aggregation at the *B. truncatus* individual scale occurred by comparing the observed distribution of trematode species within hosts with a theoretical distribution following a negative binomial distribution using a Chi-square test.

### Abundance of *B. truncatus* at each sampling site

The *B. truncatus* eDNA copy number per liter of filtered water (${C}_{L}$) estimated from the ddPCR results was used as a proxy of the abundance of *B. truncatus* at each sampling site. ${C}_{L}$ was calculated from the following equation [25]:

$$C_{L} = \frac{{\frac{{C_{rdd} *V_{e} }}{{V_{r} }}}}{{V_{w} }}$$

With ${C}_{L}$: the number of eDNA c/L for the amplified sample; ${C}_{rdd}$: the copy number per reaction volume; ${V}_{e}$: the total volume of eluted DNA after extraction; ${V}_{r}$: the volume of extracted DNA used for ddPCR reaction; and ${V}_{w}$: the total volume of filtered water.

#### Assessing the link between the relative abundance of Schistosoma quantified in co-infected *B. truncatus *and the emission status of Schistosoma cercariae by co-infected *B. truncatus*

To determine whether the dominance status of *Schistosoma* species in terms of percentage of reads of these species within *B. truncatus* individuals co-infected with other trematode species can be used as a proxy of *Schistosoma* cercarial release, we performed a generalised linear model (GLM) using the package *stats* version 4.3.1 implemented in R. A subset from our dataset that contained 14 *B. truncatus* individuals from the field work and from the experimental infection were used for this analysis. These individuals were all emitters, co-infected and harboured at least *S. haematobium* or *S. bovis*. We set the emission status (i.e. 0 and 1: non-emitting and emitting *Schistosoma* sp.) as the dependent variable and the relative abundance of the respective *Schistosoma* species in terms of percentage of sequences compared to the other co-infecting trematode species as explanatory variable. The model was built assuming a quasibinomial distribution of the data.

#### Effect of trematode species richness, co-infection rates and host abundance on the average dominance of schistosomes

To test our initial hypotheses, we ran three independent generalized linear mixed models (GLMM) using the package *lme4* version 1.1–33 implemented in R to infer the effect of (i) the overall trematode species richness using *B. truncatus* as an intermediate host at the population level, (ii) the co-infection rate among the infected *B. truncatus* snails, and (iii) the abundance of *B. truncatus;* on the average dominance of schistosomes within host populations (i.e., the relative abundance of *S. haematobium* or *S. bovis* in terms of percentage of sequences compared to the other co-infecting trematode species). Only naturally infected *B. truncatus* from the field were considered in this analysis, excluding *B. truncatus* from the experimental infection. To account for pseudo-replication, we set the sampling site as a random factor. For these analyses, the co-infection rate variable was transformed into two categories (i.e. < 50% and > 50% co-infection among infected individuals). The *B. truncatus* abundance variable was also transformed into two categories (i.e. < 200 and > 200 ${C}_{L}$) to compare sites at low and medium to high density according to Mulero et al. 2020. The variable “trematode species richness” (that uses *B. truncatus)* was coded as the number of trematode species other than *Schistosoma* which use local *B. truncatus* population at the site level. We assumed a binomial error term in the three models tested.

## Results

### Mathematical model

**Fig. 2:**
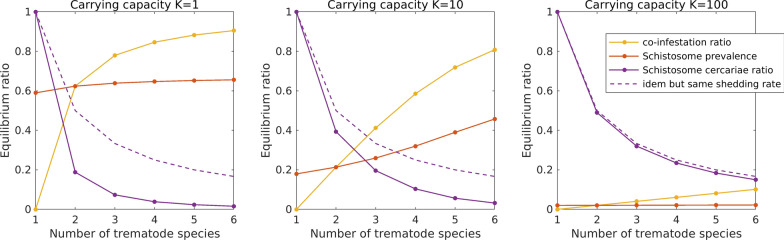
Co-infection ratio, *Schistosoma* prevalence, and *Schistosoma* cercariae ratio, for three values of the carrying capacity. The co-infection ratio is the ratio between the number of co-infected *Bulinus truncatus* and the total number of infected *B. truncatus*. The *Schistosoma* cercariae ratio is the ratio between the number of *Schistosoma* cercariae and the total number of cercariae. The dashed purple line indicates the *Schistosoma* cercariae ratio in case the *Schistosoma* shedding rate is the same as for the other trematode species. The reduction of the *Schistosoma* cercariae ratio (difference between full and dashed purple line) is due to coinfections and to the inferior competitivity of *Schistosoma*. The carrying capacity *K* differs by factors of 10 of the snail population

Our mathematical model indicates that, for a fixed carrying capacity of *B. truncatus* (either small or large), a local increase in the number of competitive trematode species using *B. truncatus* as intermediate host leads to an increase in the co-infection rate within individual snails and ultimately to a decrease in the number of *S. haematobium* cercariae produced (Fig. 2). Moreover, an increase of carrying capacity of *B. truncatus* leads to a decrease of the proportion of co-infected snails and an increase in the proportion of *Schistosoma* cercariae (Fig. 2). In other words, an increase in the number of competitive trematode species using *B. truncatus* as intermediate host can reduce the production of *S. haematobium* cercariae through antagonist interactions and particularly so when *B. truncatus* populations are small. An increase in *B. truncatus* populations mitigates the effect of antagonist interactions by reducing the coinfection rate among snail hosts.

### Empirical field study

Except *Bulinus forskalii* that was recorded at three sites (i.e., Ouali Diala, Guia and Khodit), *B. truncatus* was the only *Bulinus* species found during the field survey. The species identity of the 271 snails initially identified as *B. truncatus* based on their morphology, and from which total DNA was extracted, was validated by the LAMP-based diagnostic tool. The abundance of *B. truncatus* among sites assessed based on digital PCRs results varied from 41 to 2176 eDNA copies per litre filtered (Table 1).

**Table 1:** Summary of water volume filtered, *Bulinus Truncatus* abundance, *Schistosoma* prevalence in *B. truncatus* populations, trematode species richness that use *B. truncatus* populations as a host, co-infection rate, and average number of reads attributed to *Schistosoma* per site

Site	Volume of water filtered (L)	*B. truncatus* abundance (DNA copies / litre of filtered water)	Molecular prevalence of *Schistosoma* in *B. truncatus* (%)	Trematode richness using *B. truncatus* as host other than *Schistosoma* (number of species)	Rate of co-infections among infected individuals (Number of co-infected individuals / Total number of infected individuals)	Average percentage of *Schistosoma* reads in *B. truncatus* individuals infected by *Schistosoma* (%)
Ndiawara	6.5	78	0	4	0.3	0
Ouali_Diala	10	41	2.3	3	0.8	34.9
Guia_canal	5	61	23.8	3	1	20.7
Dioundou	10	75	0	0	NA	0
Fonde_Ass	10	57	0	1	0	0
Khodit	5	333	29.4	1	0.2	99.7
Mbane	2.8	104	0	0	NA	0
Saneinte	3.5	2176	8.3	2	0.2	50.1
Lampsar	18	58	6.3	1	0.2	80.6

**Fig. 3:**
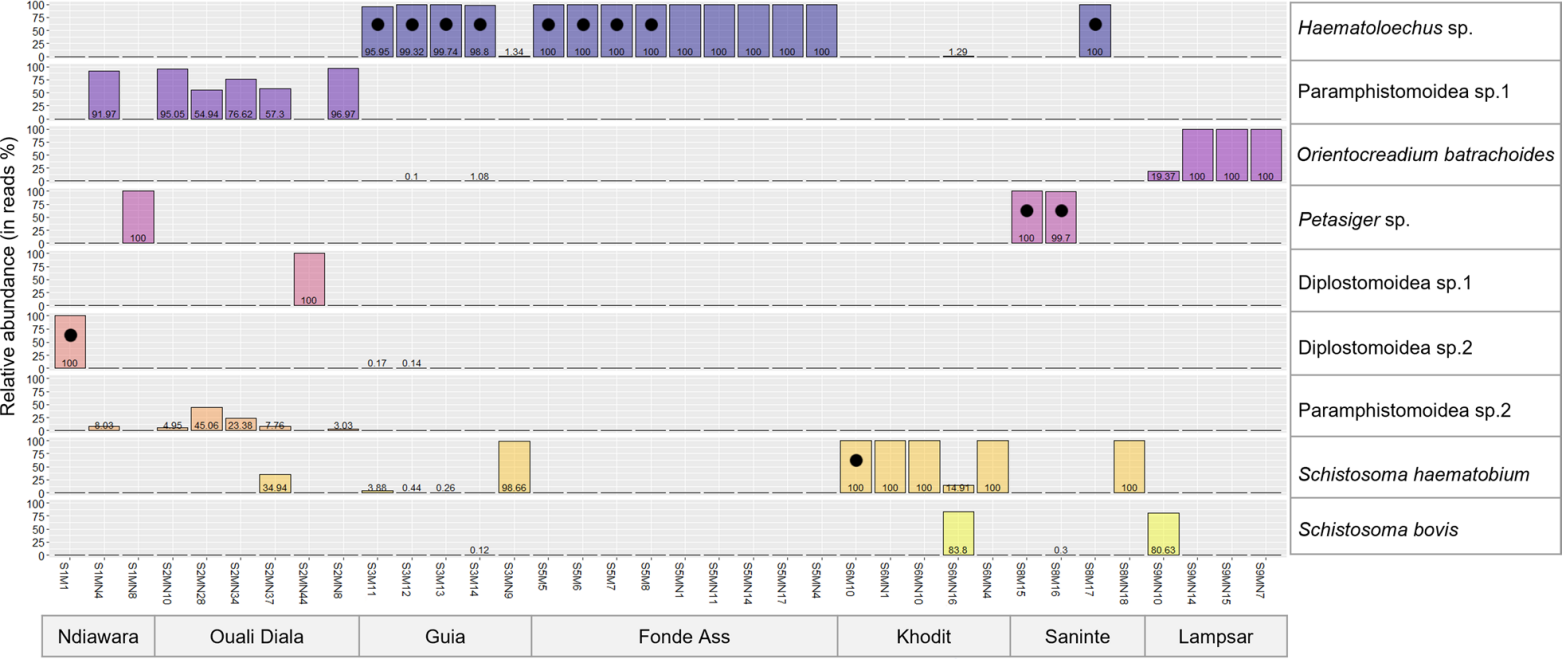
Relative abundance of trematode species in percentages of obtained sequenced reads exploiting infected *Bulinus truncatus* individual. Infected snails (in columns) are grouped by site. The black dots correspond to the species of trematode emitted by the emitting snails (confirmed by SANGER sequencing and BLAST assignation)

In addition to *S. haematobium* and its sister species *S. bovis*, we identified nine trematode species associated with *B. truncatus* populations. Nine trematode species were sampled during our fieldwork including *Haematoloechus* sp., Paramphistomoidea sp.1, *Orientocreadium batrachoides*, *Petasiger* sp., Diplostomoidea sp.1, Diplostomoidea sp.2, and Paramphistomoidea sp.2 (Fig. 3), and two additional trematode species were detected during the monitoring of experimentally infected *B. truncatus* individuals, including *Apharyngostrigea pipientis* and an *Echinostoma* sp. (Additional file 4). An additional species (i.e., Paramphistomoidea sp. 3) was found associated to two *B. truncatus* individuals from the field that were used for the experimental infection (Additional file 4) under the appearance of cysts attached on their shell (despite washing) but with no apparent shedding from snails. The number of trematode species other than S*. haematobium* and *S. bovis* using the *B. truncatus* population at each site varied from zero to four species and the co-infection rates among infected individuals from 0 to 100% (Fig. 3; Table 1). The distribution of the number of trematode species per *B. truncatus* individual from the field did not differ significantly from a theoretical negative binomial distribution hence indicating a classical parasite aggregation pattern in *B. truncatus* populations (Additional file 5).

In the water samples we detected a total of 20 trematode species, seven of which were also detected in *B. truncatus*. Conversely, two trematodes detected in *B. truncatus* were not detected from the water samples. *Schistosoma haematobium* and/or *S. bovis* were detected from eDNA water filtrates in five on the nine sampled sites (Additional file 6).

During all the molecular analysis steps, all the negative and positive controls behaved as expected. The prevalences of trematodes including that of schistosomes in *B. truncatus* populations, assessed from our molecular diagnostic approach, were higher than the prevalences measured by the emission method only (Additional file 7).

Whether from field or experimental infection, the emitting *B. truncatus* infected by *Schistosoma* species and by at least one other species of trematode, *Schistosoma* species are shed only when they are dominant in terms of relative sequenced reads abundance compared to that of the other co-infecting trematode species (Additional file 8). Moreover, in all but one case, the haplotype of the trematode species emitted corresponds to the most abundant haplotype obtained from NGS sequencing in co-infected *B. truncatus* (Fig. 3; Additional file 4).

Both the local richness of trematode species exploiting *B. truncatus* population (Z value = -2.17; *P*-value = 0.029; AIC = 14.9) and the coinfection rate (Z value = -2.32; *P*-value = 0.02, AIC = 17.4) have a significant negative effect on *Schistosoma* spp. dominance based on our GLMM models. Conversely, the local abundance of *B. truncatus* positively and significantly affects *Schistosoma* spp. dominance (Z value = 1.98; *P*-value = 0.047, AIC = 20.1). Based on the comparison of AICs obtained from the three statistical models ran, the model accounting for the local richness of trematode species exploiting *B. truncatus* population is the best supported which strengthens the idea that this factor is the most important in explaining *Schistosoma* spp. dominance. At site level, the co-infection rate among infected snails tended to increase when the total trematode species richness exploiting the *B. truncatus* population increased, although the correlation was not significant (Table 1). Conversely, this same co-infection rate tended to decrease when the abundance of *B. truncatus* increased (Table 1).

## Discussion

Understanding the ecological mechanisms that drive the circulation of parasites is primordial to better identify transmission site, better assess transmission risks and guide strategies to fight against parasites and the associated diseases while preserving the integrity of the socio-ecosystem health [32, 33]. In this context, the ecology of transmission of *Schistosoma* species, and the impact of human activities on the circulation of these parasites, have received considerable attention and particularly so in the last decade [34, 35].

The presence and abundance of compatible snail hosts in a given system are determining factors in the establishment and circulation of *Schistosoma* parasites, particularly as they modulate the rate of contact between the parasites released into the system and the hosts (i.e., encounter filter [36, 37]).

In the present study we theoretically and empirically show that reducing snail host abundance also hamper the circulation of *S. haematobium* indirectly by promoting snail co-infections with other potentially more competitive trematode species and hence a reduction of *S. haematobium* cercariae produced by local *B. truncatus* populations. This competition effect on the circulation of *S. haematobium* becomes negligeable when *B. truncatus* populations are abundant. In other words, the size of local snail host populations for parasites of the genus *Schistosoma* may predict the risk of transmission for definitive vertebrate hosts including humans because it determines not only the encounter filter, but also modulates the ‘competence’ (sensu* largo*) of the locally established snail hosts by influencing their probability of being co-infected with other competitive trematode species (i.e., compatibility filter; [36, 37]). This result is based on the generally acknowledged assumption that *Schistosoma* species are bad to moderate competitors [13, 15]. Although we could not empirically assess the hierarchical rank of competitive ability of each trematode detected in *B. truncatus* populations during our field survey we can expect that at least some of these outcompete or even predate *S. haematobium*, particularly those known to produce rediae during their intra-mollusc parasitic stage such as *Petasiger* sp.

Coinfections are rarely observed in the field which could suggest that the effect of the resulting antagonistic interactions between co-occurring parasites could be of relatively weak importance [15]. We here argue that coinfections are likely to be generally underestimated. In particular, we believe that traditional techniques to detect trematodes that develop within their snail hosts via dissection or induced cercarial emission, might lead to an underestimation of coinfection rates. This might be particularly true when *Schistosoma* species are present in the form of degraded, or even invisible traces, resulting from competition or predation induced by other trematodes species. In this regard, high throughput next generation sequencing approaches such as metabarcoding used in this study appear promising to better assess coinfection rates and overall trematode communities within natural snail host populations [38]. These metabarcoding approaches are the ‘interspecific equivalent’ of the genotyping approaches that have revealed the initially unsuspected intraspecific diversity of parasites within their hosts in the past decades [39–41].

Our model also points toward the fact that the intensity of such within-snail host competitive effects on *S. haematobium* cercariae production increases with the number of trematode species using the targeted *B. truncatus* population. This theoretical result is also supported by our empirical study. It is interesting to note that the presence of a single highly competitive trematode species at high abundance at a given transmission site and using a given population of *B. truncatus*, would locally result in a similar decrease in the amount of cercariae of *S. haematobium* co-occurring at this site. In fact, biological control strategies based on the dissemination of farmable competitive trematode species that use snail vectors of parasites of the genus *Schistosoma* at active transmission sites have been proposed in the past [42]. However, this situation, if not artificially maintained, is generally uncommon *in natura*. Conversely, several studies, including ours, indicate that parasites of the genus *Schistosoma* naturally cooccur frequently with several trematode species that use *Schistosoma* snail intermediate hosts in the field [13]. Since trematode species composition is determined by the composition of vertebrate communities locally established either temporarily or permanently [43], maintaining vertebrate host diversity that supports trematode diversity in local snail populations could help to reduce the transmission of *Schistosoma* species. For this effect to be sustainable over time, the trematodes released by their vertebrate definitive hosts must complete their entire life cycle locally, which also implies the maintenance of other compartments of biodiversity, including numerous vertebrates (e.g., fish and amphibians) and invertebrates (e.g., aquatic insects). Unfortunately, our ecological knowledge of most trematodes is sorely lacking, and their life cycles are still poorly documented. Huge works remain to be done to characterise both the fauna involved in supplying trematodes that interact with species of the genus *Schistosoma* in their intermediate hosts, and the fauna involved in maintaining the local life cycles of these trematodes. We believe that the newly available metabarcoding tools such as used in the present study [20, 38, 44], combined with the implementation of large and well documented sequence datasets, provide a promising avenue to characterise trematode life cycles.

Our study suggests that maintaining high levels of biodiversity in freshwater aquatic ecosystems could help reducing the transmission of parasites of the genus *Schistosoma*. Several synergetic effects of biodiversity could reduce the circulation of these parasites [33]. For instance, several organisms including fish, crustaceans, and oligochaetes can predate free-living stages of *Schistosoma* parasites and hence reduce their abundance and therefore the risk of infection for mammals [45]. However, the negative effect of local biodiversity on the circulation of *Schistosoma* species is all the greater when host snail populations are small. Eradication of snail populations that host *Schistosoma* species is generally considered as the best alternative to fight against schistosmiasis and is one of the recommendations of the WHO [46]. Although this strategy is theoretically efficient at short term, its deployment on a large spatial and temporal scale is questionable for several reasons. First, the molluscicides commonly used and spread into aquatic ecosystems are sometimes poorly accepted by Human communities using these ecosystems for water supply. Second, the effects of molluscicides on the overall biodiversity associated with aquatic environments are still poorly understood [47–50]. Finally, while these products are efficient against snail communities established locally, they do not prevent the recolonization of these species after treatment. In this context, and alternatively to the application of chemical-based molluscicide, several environment-based strategies aiming at reducing snail hosts abundance while providing services to local human populations are emerging, such as the introduction of edible predators of snail hosts (e.g., crayfish) or the extraction of specific aquatic plants that serve as refuges for aquatic snails to produce compost [51, 52]. Combined with massive drug administration campaign in human populations and the application of the WASH protocol [46], these guided sustainable strategies of reduction or eradication of aquatic snail host populations that constitute intermediate hosts for *Schistosoma*, generally lead to an important reduction in the rate of re-infection and prevalence of schistosomiasis in neighbouring human populations [53]. We here argue that the implementation of sustainable strategies to preserve the biodiversity associated with aquatic ecosystems could be an additional and potentially low-cost means of significantly reducing the prevalence and intensity of bilharziasis, without necessarily eliminating totally host snail populations. These strategies are in line with the need for concrete applications of the One Health concept, particularly recently applied in the case of aquatic parasites including schistosomes [54], and the objectives established by the WHO to reduce the prevalence and intensity of schistosomes below a threshold below which these diseases can no longer be considered a public health problem [46].

## Conclusions

Biodiversity can buffer the circulation of pathogens, including parasites with complex life cycles such as *Schistosoma* species through several ecological processes. Our study theoretically and empirically highlights the importance of high levels of trematode biodiversity in promoting co-infections between the moderately competitive *Schistosoma* species and other trematode species within their snail hosts, hence limiting the production of *Schistosoma* cercariae and the circulation of these species. Species of trematodes that use the same snail hosts as *Schistosoma* species are numerous. This suggests that interactions between schistosomes and other trematodes are important natural ecological processes that need to be studied in greater depth and considered when fighting against the circulation of *Schistosoma* species. Importantly however, we also demonstrate this effect of biodiversity on the circulation of *Schistosoma* species mainly holds when snail hosts populations are small and tends to diminish when snail hosts populations increase in abundance. This result provides further support for the idea that it is important to regulate the population size of snail hosts of *Schistosoma* to control the circulation of these parasites, without necessarily having to fully eradicate these populations by unsustainable means.

## Supplementary Information

Additional file 1

Additional file 2

Additional file 3

Additional file4

Additional file 5

Additional file 6

Additional file 7

Additional file 8

Additional file 9

Additional file 10

Additional file 11

Additional file 12

## Data Availability

All data generated or analysed during this study are included in this published article [and its supplementary information files]. This project obtained from the Nagoya office in Senegal, an exemption from authorization of access and use of genetic resources (number: 001339 of November 15, 2021, reference: V/L du 28 octobre 2021) by the Competent National Authority (Directorate of National Parks of Senegal).
