# Supplementary material for: The abundance of snail hosts mediates the effects of antagonist interactions between trematodes on the transmission of human schistosomes
Source: Infect Dis Poverty. 2024 Sep 10;13:65. doi: 10.1186/s40249-024-01232-1 (PMC11386086; doi:10.1186/s40249-024-01232-1)
Supplement: Supplementary file 1 — Additional file 1 [file 40249_2024_1232_MOESM1_ESM.docx]

## Supplementary material

In this appendix we provide an in-depth description of the model equations and our simulations.

We start with the model for a single trematode species, and then present the extension to two and more trematode species. The model for the general case is rather complex, and we explain how we use a number of assumptions to simplify the computation of the model equilibrium. Finally, we provide the details to generate Figure2 of the main text.

**Model with one trematode species**


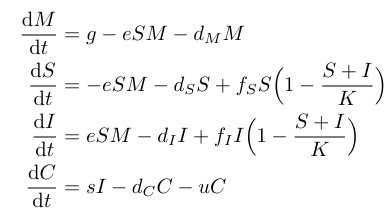
The model tracks the dynamics of the aquatic ecosystem consisting of miracidia, susceptible and infected snails, and cercariae. We denote their densities by the variables *M(t)*, *S(t)*, *I(t)* and *C(t)* respectively. The dynamics are described by the following set of ordinary differential equation :

(S1)

The meaning of the model parameters is as follows: *g* : input rate of miracidia; *e* : infection rate of susceptible snails by miracidia ; *f_S_*, *f_I_* : fitness of susceptible and infected snails; *K* : carrying capacity of snail population; *s* : shedding rate of infected snails; *u* : uptake rate of cercariae by mammals; *d_M_*, *d_S_*, *d_I_* , *d_C_* : loss rates of miracidia, susceptible snails, infected snails, cercariae.

Because infection reduces fitness and increases mortality, we require that *f_I_* < *f_S_* and *d_I_* > *d_S_*. For simplicity we take the extreme case and set *f_I_* = 0 and *d_S_* = 0. Note that the model does not describe the complete trematode life cycle. Rather, the model starts from a given exposure to miracidia (parameter *g*) and predicts the cercariae density (variable *C*), or equivalently the uptake of cercariae by mammal hosts (equal to *uC*). To simplify the study of the model, we focus on the equilibrium of the model dynamics (Additional _File_9_(Figure_S1)).

**Model with two trematode species**


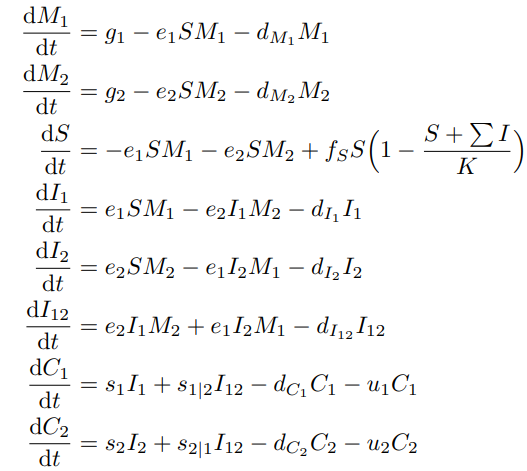
Next, we extend model equations (S1) to two trematode species infecting a snail population. We call these two species *1* and *2*. Compared to the previous model, we extend the list of variables as follows: miracidia *M_1_* and *M_2_*, susceptible *S*, infected *I_1_*, *I_2_* and co-infected *I_12_*, and cercariae *C_1_* and *C_2_*. The equations are :

(S2)

The meaning of the model parameters is as before, but now with the indices *1* and *2* referring to the trematode species. For the shedding rates, *s_1_* is the shedding rate of cercariae of species *1* by a snail infected by species *1* only, whereas *s_1|2_* is the shedding rate of cercariae of species *1* by a snail infected by both species. Similar definitions hold for *s_2_* and *s_2|1_*. We assume that the two trematode species only differ in their shedding rates. Note that the term “shedding” used in the model reflect the transition from the day when snails become infected to the release of cercariae, and hence also includes the development of the trematodes within the snail hosts. Taking species *1* as a *Schistosoma* species, we model its inferior competitivity by requiring that *s_1|2_* < *s_2|1_* (i.e., co-infected snails preferentially shed cercariae of species *2*). For simplicity, we set *s_1|2_* = 0 and *s_2|1_* = *s_2_*. In other words, snails that are infected by both species do not shed *Schistosoma* cercariae, and they shed cercariae of the other species at the same rate as a snail not co-infected by *Schistosoma*. Apart from this difference in shedding rates, we assume the two trematode species to be equivalent for all other model parameters (Additional _File_10_&_11_(Figure_S2_and_S3)).

**Arbitrary number of trematode species**

In principle the models for one and two trematode species, equations (S1) and (S2), can be readily extended to an arbitrary number of trematode species. However, the resulting model is rather complex. This can be seen by counting the number of dynamical variables. For the model with one trematode species, there are 4 variables (*M*, *S*, *I*, *C*). In the case of two trematode species, there are 8 variables (2**M*, 1**S*, 3**I*, 2**C*). In the general case of n trematode species, there are 2*^n^* +2*n* variables (*n***M*, 1**S*, (2*^n^*-1)*I, *n***C*). For example, for *n* = 10 there are 1044 variables, and hence we would have to solve a system of 1044 equations to find the equilibrium densities.


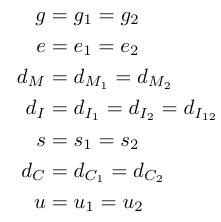
The model complexity, and the computation of the equilibrium, can be drastically reduced by two assumptions, which we illustrate for the case of two trematode species. First, we take the *Schistosoma* species (the first species) to be an inferior competitor. We assume that snails that are infected by both trematode species only shed cercariae of the second species, with the same rate *s_2_* as for snails that are only infected by the second species (i.e., *s_1|2_* = 0 and *s_2|1_* = *s_2_*). Second, we assume that the two trematode species have the same value for all other model parameters. That is, we set:


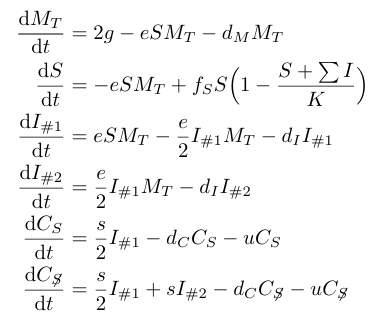
Introducing these two assumptions into equations (S2), we see that variables *M_1_* and *M_2_* and also variables *I_1_* and *I_2_* have exactly the same dynamics. This implies that at equilibrium (when the initial condition no longer have any effect) we have *M_1_* = *M_2_* and *I_1_* = *I_2_*. We can then simplify the model by introducing the total miracidia density *M_T_* = *M_1_* +*M_2_*, the density of snails infected by one trematode species *I_#1_* = *I_1_*+*I_2_* and the density of snails infected by two trematode species *I_#2_* = *I_12_*. We also make explicit in our notation that the first trematode species is the *Schistosoma* species and that the other species is not by writing *C_S_* = *C_1_* and *C_~~S~~_* = *C_2_*. The dynamical equation become :

(S3)

Note that in this way we have reduced the number of equations from 8 to 6.

The same approach can be applied to the general model of *n* trematode species. The trick is again to work with the total miracidia density *M_T_* and with the densities of snails infected by one, two, three, ... trematode species *I_#1_*, *I_#2_*, *I_#3_*, ... The reduced dynamics are :


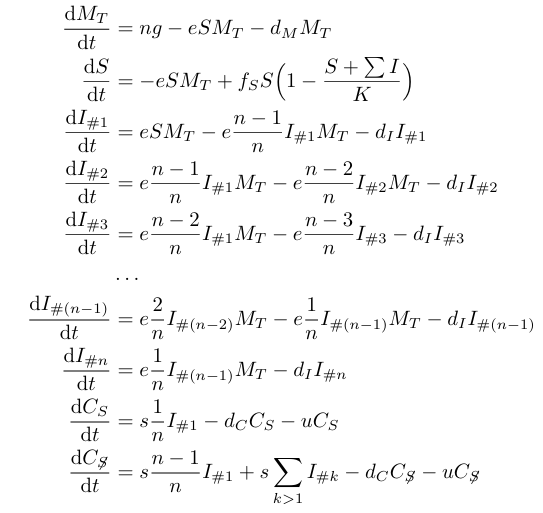


(S4)

The prefactors of the terms in these equations are easy to understand. For example, the term $e\frac{n-2}{n} I_{\#2}M_{T}$ appearing in the equations for $\frac{{dI}_{\#2}}{d_{t}}$ (with a minus sign) and for $\frac{{dI}_{\#3}}{d_{t}}$ (with a plus sign) corresponds to an infection by a third trematode species. The prefactor $\frac{n-2}{n}$ excludes infections by one of the two trematode species by which the snail is already infected. As another example, the term $s\frac{1}{n} I_{\#1}$ in the equation for $\frac{{dC}_{s}}{d_{t}}$ corresponds to the shedding of *Schistosoma* cercariae. This can only originate from snails that are infected by a single trematode species, hence the factor *I_#1_ ,* and the infected trematode should belong to the *Schistosoma* species, hence the factor $\frac{1}{n}$ .

After the reduction there are *n*+4 dynamical variables. This is a drastic simplification compared to the number of variables before reduction. For example, for *n*=10 trematode species, there are only 14 equations in the reduced system, instead of the 1044 equations in the original system.

**Parameter values used in main text**

To generate Figure2 of the main text, we simulate the model equations until reaching equilibrium. We used the following parameter values:

| *n* | number of trematode species | 1 to 6 | / |
| --- | --- | --- | --- |
| *g* | input rate of miracidia per trematode species | 0.2 | *d_U_*/*t_U_* |
| *e* | infestation rate of snails by miracidia | 0.01 | 1 / (*d_U_ t_U_*) |
| *f_S_* | fitness of non-infected snails | 3.0 | 1 / *t_U_* |
| *K* | carrying capacity of snail population | 1, 10, 100 | *d_U_* |
| *s* | shedding rate by infected snails | 2.0 | 1 / *t_U_* |
| *u* | uptake rate of cercariae by mammals | 1.0 | 1 / *t_U_* |
| *d_M_* | loss rate of miracidia | 0.01 | 1 / *t_U_* |
| *d_I_* | loss rate of infected snails | 0.1 | 1 / *t_U_* |
| *d_C_* | loss rate of cercariae | 0.1 | 1 / *t_U_* |

The last column gives the units in which the parameters are expressed. For example, the time unit *d_t_* could be month, and the density unit *d_u_* could be number of individuals per square meter. Note that the model results do not depend on the choice of these units.


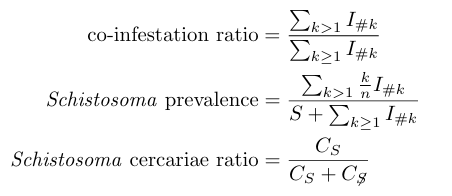
Finally, the plotted variables are computed as follows:

We provide Matlab code to perform the different computations. In particular, running the script divpara_main.m generates Figure2 of the main text. This script calls the function divpara_rhs.m to evaluate the right-hand side of the model equations, i.e., the function implements model equations (S4).
