# Supplementary figures and images for: The abundance of snail hosts mediates the effects of antagonist interactions between trematodes on the transmission of human schistosomes
Source: Infect Dis Poverty. 2024 Sep 10;13:65. doi: 10.1186/s40249-024-01232-1 (PMC11386086; doi:10.1186/s40249-024-01232-1)

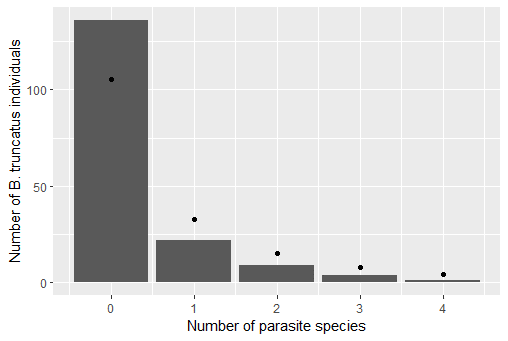

Supplement: Supplementary file 5 — Additional file 5 [file 40249_2024_1232_MOESM5_ESM.png]

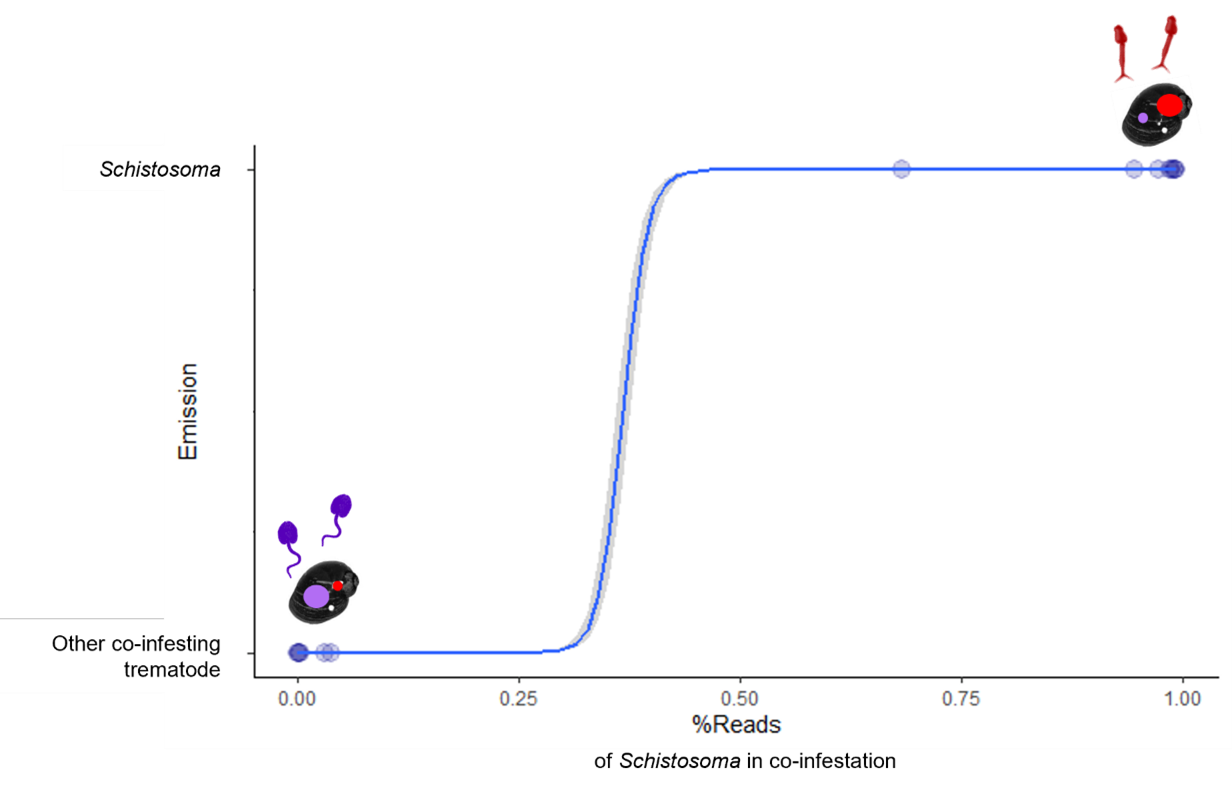

Supplement: Supplementary file 8 — Additional file 8 [file 40249_2024_1232_MOESM8_ESM.png]

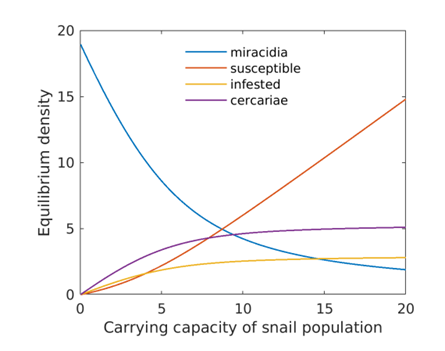

Supplement: Supplementary file 9 — Additional file 9 [file 40249_2024_1232_MOESM9_ESM.png]

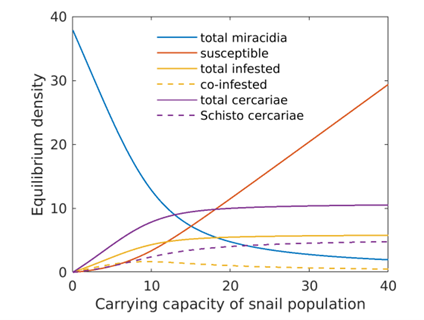

Supplement: Supplementary file 10 — Additional file 10 [file 40249_2024_1232_MOESM10_ESM.png]

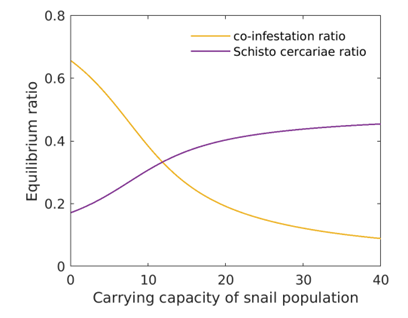

Supplement: Supplementary file 11 — Additional file 11 [file 40249_2024_1232_MOESM11_ESM.png]

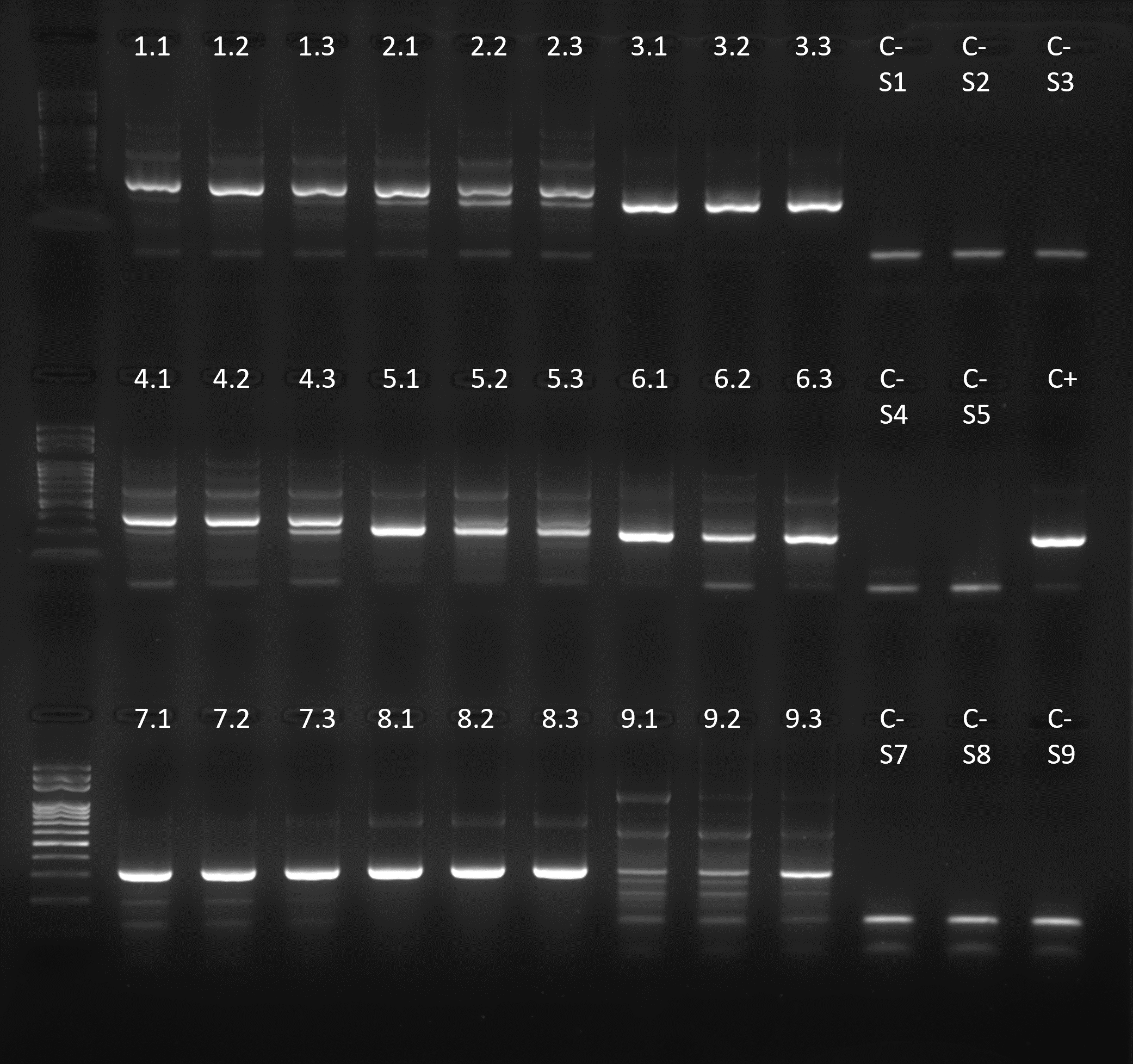

Supplement: Supplementary file 12 — Additional file 12 [file 40249_2024_1232_MOESM12_ESM.png]
